# Pulmonary Hypertension in a Patient With Kartagener’s Syndrome and a Novel Homozygous Nonsense Mutation in CCDC40 Gene: A Case Report

**DOI:** 10.3389/fmed.2022.860684

**Published:** 2022-03-30

**Authors:** Hai-Long Dai, Duolao Wang, Xue-Feng Guang, Wei-Hua Zhang

**Affiliations:** ^1^Key Laboratory of Cardiovascular Disease of Yunnan Province, Department of Cardiology, Clinical Medicine Center for Cardiovascular Disease of Yunnan Province, Yan’an Affiliated Hospital of Kunming Medical University, Kunming, China; ^2^Department of Clinical Sciences, Liverpool School of Tropical Medicine, Liverpool, United Kingdom

**Keywords:** Kartagener’s syndrome, genetics, mutation, CCDC40, case report

## Abstract

Kartagener’s syndrome is a subgroup of primary ciliary dyskinesia (PCD), a genetically heterogeneous condition characterised by sinusitis, bronchiectasis, and situs in versus. Genetic testing has importance for their diagnosis. Here, we report a chinese patient with Kartagener’s syndrome. Transthoracic echocardiography showed severely elevated right ventricular systolic pressure. Right heart catheterisation demonstrated a pre-capillary pulmonary hypertension. Whole-exome sequencing indicated that she had a novel homozygous nonsense mutation, c.2845C > T, p.Gln949*, in exon 18 of CCDC40 and a heterozygotic mutation, c.73G > A, p.Ala25Thr, in exon 1 of DNAH11. She was diagnosed as Kartagener’s syndrome with pulmonary hypertension. Her symptoms improved significantly by treatment of antibiotics, expectorant drugs, bronchodilators, and oxygen therapy treatment. Our findings extend the mutation spectrum of CCDC40 gene related Kartagener’s syndrome, which is very important for gene diagnosis of the disease.

## Introduction

Primary ciliary dyskinesia (PCD), also known as immobile cilia syndrome, is a very rare disease. One case was found in 26000-40000 live births, representing the clinical and genetic heterogeneity group of respiratory ciliopathies, with reduced airway mucociliary clearance (1). About 50% of cases show clinical triad of Kartagener’s syndrome (KS), an important and rare subgroup of PCD, more common among people with consanguineous marriages. The syndrome includes the clinical triad of chronic sinusitis, bronchiectasis, and situs in versus Siewert first reported those combination in 1904, then Manes Kartagener identified this clinical triad as a unique congenital syndrome in 1933 (1, 2). In the embryonic stage, the position of the organ is determined by the uniform beating of the ciliary body, but in KS, due to the movement disorder of the ciliary body, the heart and other organs cannot move to the left, resulting in dextrocardia and situs inversus.

Genetic testing have importance for a PCD/KS diagnosis (3). Next generation sequencing increased gene discovery, with more than 40 gene mutations reported to cause PCD (4). The

CCDC40 (coiled-coil domain containing protein 40) gene is essential for ciliary motor function and left–right axis formation. Mutations in the CCDC40 gene result in the dislocation of central microtubule pair and the assembly defect of internal dynein arm and dynein regulatory complex, resulting in hypomotile or immotile cilia (5). The DNAH11 (dynein axonemal heavy chain 11) gene encodes a ciliary outer dynein arm protein required for cilia motility (6–8).

Here, we reported a rare case of pulmonary hypertension (PH) caused by KS, and whole exome sequencing (WES) was used to study gene mutation.

## Case Description

A 26-year-old woman with a 20-year history of shortness of breath on exertion was admitted to our hospital. Since childhood she had recurrent episodes of respiratory tract infections with sinusitis. Six years ago, she was diagnosed with pulmonary arterial hypertension and dextrocardia only by echocardiography (pulmonary artery systolic pressure 133 mmHg). She has been taking bosentan 62.5 mg twice daily and intermittent diuretic therapy for the past 6 months. She never smoked and had no history of illicit drug or alcohol use. The parents of the patient were consanguineous marriage. At admission, the patient’s temperature was 36.3°C, her BP was 104/61 mmHg, respiratory rate was 19 breaths/min, and pulse oximetry was 70% on room air. Physical examination revealed apex beat on the right 5th intercostal space in mid-clavicular line with audible heart sounds, and a few moist rales in the lungs. No pathologic heart sounds were detected. Cyanosis of the lips and nails.

Laboratory data on admission showed a haemoglobin level of 15.4 g/dL (normal, 11.0–15.0 g/dL), high sensitivity C-reactive protein level of 37.64 mg/L (normal value, < 3 mg/L), N-terminal B-type natriuretic peptide precursor (NT-proBNP) level of 285 ng/L (normal value, < 125 ng/L), and a small amount of gramme-negative bacilli were detected in sputum smear. The following laboratory results were normal/negative: WBC count, BUN, creatinine, liver chemistry tests, thyroid functions, urine analysis, levels of troponin I level, D-dimer, HIV, hepatitis panel, the antibody of rheumatic disease, and microbiology and mycological culture of sputum. Her arterial blood gas analysis showed pH of 7.419, SO2 of 66.5%, pO2 of 36.4 mm Hg, and pCO2 of 38.2 mm Hg.

**FIGURE 1:**
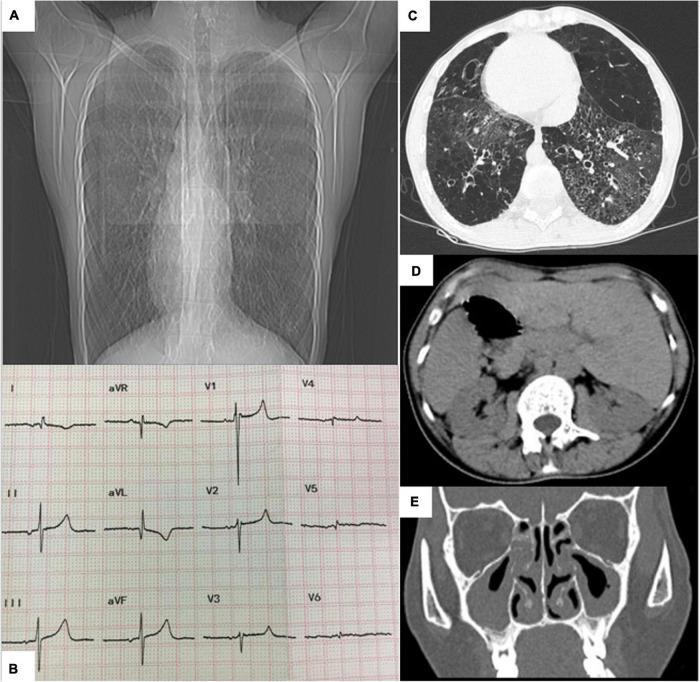
**(A)** Chest radiograph showing dextrocardia. **(B)** Electrocardiogram showing dextrocardia with tall R waves in lead V1 and absent R in V6. **(C)** Chest CT showing bronchiectasis. **(D)** Abdomen CT image showing liver on the left, stomach and spleen on the right. **(E)** CT of sinuses showing mucosal thickening.

Chest radiography revealed a flipped cardiac silhouette with the apex of the heart at the right thorax (Figure 1A). Electrocardiogram results showed dextrocardia with tall R waves in lead V1 and absent R in V6 (Figure 1B). CT scan of the chest and abdomen that showed bronchiectasis with infection, emphysema and mosaic attenuation (Figure 1C), liver on the left and spleen on the right (Figure 1D). CT of sinuses showed mucosal thickening in maxillary sinus, sphenoid sinus and ethmoid sinus (Figure 1E). Transthoracic echocardiography showed dextrocardia, normal cardiac chamber size, hypertrophied right ventricle free wall, mild tricuspid regurgitation, and severely elevated right ventricular systolic pressure (90 mmHg). Her pulmonary function tests showed decreased FVC (1.15 L or 30.8% predicted), decreased FEV1 (0.55 L or 17.0% predicted), an decreased Tiffeneau-Pinelli index (FEV1/FVC) (0.48 with 0.88 predicted), a total lung capacity (TLC) of 71.1% predicted, decreased DLCO (8.3% predicted) and RV/TLC of 0.61, all suggestive of mixed ventilation dysfunction, diffuse function impairment, and emphysema.

Right heart catheterization (RHC) was performed under oxygen inhalation (3L/min), which demonstrated a pre-capillary PH, right atrial pressure was 6 mmHg; pulmonary artery (PA) pressure was 46/21/32 mm Hg; PA wedge pressure was 10 mm Hg; cardiac index was 2.78 L/min/m^2^, and pulmonary vascular resistance was 5.34 Wood units.

**FIGURE 2:**
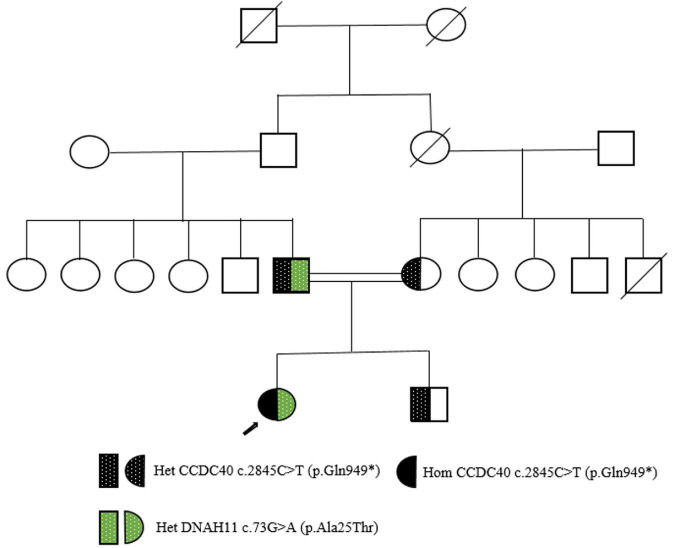
Pedigree of a Han-Chinese patient with Kartagener’s syndrome. Square represents male; circles represent females; arrow presents the proband; double lines indicate consanguinity. Het, heterozygous; Hom, homozygous.

**FIGURE 3:**
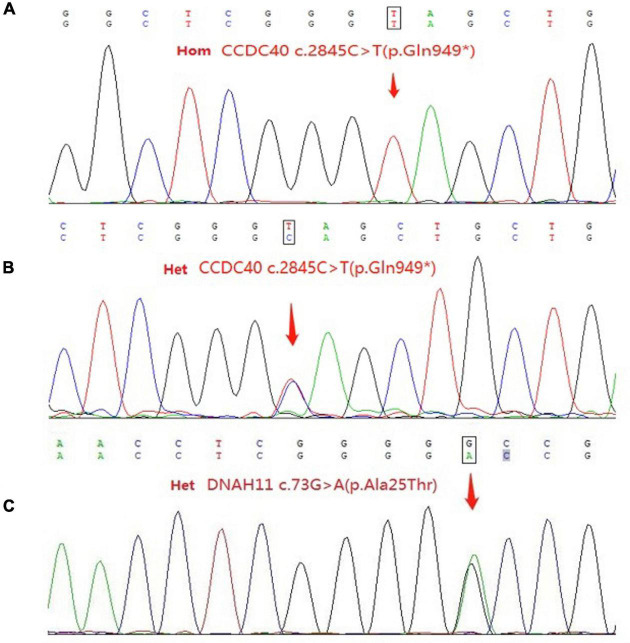
**(A)** Sequence of the homozygous c.2845C > T (p.Gln949*) variant in the patient. **(B)** Sequence of the heterozygous c.2845C > T (p.Gln949*) variant among relatives of patient. **(C)** Sequence of the heterozygous c.73G > A (p.Ala25Thr) variant in the patient and her father. Het, heterozygous; Hom, homozygous.

This patient from a consanguineous parents (Figure 2), we performed whole-exome sequencing by next-generation sequencing in this patient, her parents and her brother (Figure 3) that revealed a novel homozygous nonsense mutation in the CCDC40 gene (NM_017950.4: c.2845C > T: p.[Gln949*]) in this patient, which results in early termination of protein translation, heterozygous state in her parents, and a reported heterozygotic missense mutation in the DNAH11 gene (NM_001277115.2: c.73G > A:p.[Ala25Thr]) may result in the protein dysfunction (3), suggestive of PCD. And, no pathogenic gene of PH was found.

This patient was diagnosed as KS and PH related to pulmonary diseases. She was started on expectorant drugs, bronchodilators, oxygen therapy, chest physiotherapy for airway clearance, and anti-infective therapy with meropenem and ceftazidime, which have a broad spectrum of antimicrobial action, including the most clinically important microorganisms: Gramme-positive, Gramme-negative, aerobic, and anaerobic. After 2 weeks of therapy, her symptoms and chest CT improved significantly, pulse oximetry saturation was 90% while oxygen therapy (3L/min), and she was discharged. At the 12-month follow-up, the patient was doing well.

## Discussion

Primary ciliary dyskinesia is an inherited, genetically and clinically heterogeneous disorder which causes ciliary dyskinesia, which leads to the clinical phenotype of chronic sino-pulmonary disease and a rare cause of male infertility (9). KS is a type of PCD, which is a mirror-image reversal of the heart and other internal organs.

Laboratory screening tests for PCD exhaled nasal nitric oxide level determination and saccharin test for assessing nasal epithelial mucociliary function, but saccharin test is no longer advocated. High-speed video microscopy for evaluating ciliary beat frequency and pattern, transmission electron microscopic, immunofluorescence of ciliary proteins and electron microscopy tomography for detecting ultrastructural ciliary defect, and genetic testing are confirmatory laboratory tests (10, 11). Our patient presented with recurrent episodes of pulmonary infections. Imaging examination showed dextrocardia, bronchiectasis, and situs in versus. This patient had a novel homozygous variation in the CCDC40 gene, which lead to early termination of protein translation. We also found a heterozygotic missense mutation in DNAH11 gene, some sudies showed DNAH11 mutations can cause PCD and KS (12, 13). However, her father had no clinical manifestations. Thus, KS in this patient mainly explained by the CCDC40 mutation.

Pulmonary hypertension frequently complicates the course of patients with various forms of chronic lung disease (CLD)(14). These patients are classified under group-3 according to WHO-PH classification. In current guidelines, the optimal treatment of the underlying lung disease, including long-term oxygen treatment in patients with chronic hypoxaemia, is recommended in patients with CLD-PH.

At present, there is no specific therapy for KS. The main treatment principles includes chest physiotherapy, mucolytics, antibiotics and regular vaccination to prevent respiratory tract infection (15). Lung transplantation is the effective treatment for end-stage KS (16), this patient not accepted our recommendation for lung transplantation. In the future, the genetic correction of mutated PCD genes may be a potential therapeutic method (17, 18).

In conclusion, we reported that pulmonary hypertension in a patient with Kartagener’s syndrome and a novel homozygous nonsense mutation in CCDC40 gene. It is helpful for understanding this disease and expanding the mutation database.

## Ethics Statement

Ethical review and approval was not required for the study on human participants in accordance with the local legislation and institutional requirements. The patient/participant provided her written informed consent to participate in this study.

## Author Contributions

X-FG and W-HZ collected the data and participated in writing the manuscript. H-LD conceived this study and participated in writing the manuscript. H-LD, W-HZ, and X-FG participated in patient management. DW contributed to critical revision. All authors have read and approved the final manuscript.

## Conflict of Interest

The authors declare that the research was conducted in the absence of any commercial or financial relationships that could be construed as a potential conflict of interest.

## Publisher’s Note

All claims expressed in this article are solely those of the authors and do not necessarily represent those of their affiliated organizations, or those of the publisher, the editors and the reviewers. Any product that may be evaluated in this article, or claim that may be made by its manufacturer, is not guaranteed or endorsed by the publisher.
